# The impact of spatial and temporal availability of alcohol on its consumption and related harms: A critical review in the context of UK licensing policies

**DOI:** 10.1111/dar.12191

**Published:** 2014-09-04

**Authors:** John Holmes, Yelan Guo, Ravi Maheswaran, James Nicholls, Petra S Meier, Alan Brennan

**Affiliations:** 1 School of Health and Related Research, University of SheffieldSheffield, South Yorkshire, UK; 2 Alcohol Research United KingdomLondon, UK; 3 Centre for History in Public Health, London School of Hygiene and Tropical MedicineLondon, UK

**Keywords:** alcohol consumption, spatial analysis, review, alcohol outlet density

## Abstract

**Issues:**

Reviews recommend controlling alcohol availability to limit alcohol-related harm. However, the translation of this evidence into policy processes has proved challenging in some jurisdictions.

**Approach:**

This paper presents a critical review of empirical spatial and temporal availability research to identify its features and limitations for informing alcohol availability policies. The UK is used as an example jurisdiction. It reviews 138 studies from a 2008 systematic review of empirical availability research and our update of this to January 2014. Data describing study characteristics (settings, measures, design) were extracted and descriptively analysed.

**Key Findings:**

Important limitations in current evidence were identified: (i) outlet-level temporal availability was only measured in three studies, and there has been little innovation in measurement of spatial availability; (ii) empirical analyses focus on acute harms with few studies of longer-term harms; (iii) outlets are typically classified at aggregated levels with little empirical analysis of variation within outlet categories; (iv) evidence comes from a narrow range of countries; and (v) availability away from home, online availability and interactions between availability, price and place are all relatively unexamined.

**Implications:**

Greater innovation in study and measure design and enhanced data quality are required. Greater engagement between researchers and policy actors when developing studies would facilitate this.

**Conclusions:**

*Research and data innovations are needed to address a series of methodological gaps and limitations in the alcohol availability evidence base, advance this research area and enable findings to be translated effectively into policy processes.* [Holmes J, Guo Y, Maheswaran R, Nicholls J, Meier PS, Brennan A. The impact of spatial and temporal availability of alcohol on its consumption and related harms: A critical review in the context of UK licensing policies. *Drug Alcohol Rev* 2014;33:515–25]

## Introduction

Controlling the spatial and temporal availability of alcohol has been identified as a key approach for reducing alcohol-related harm [1]. Although this conclusion is supported by a large body of empirical evidence 2–5, translating that evidence into practice has proved challenging in some jurisdictions. Using the UK as an example, this paper considers future directions for empirical availability research with a view to facilitating the translation of findings into policy design and implementation. We also consider how non-translational research in this area can be advanced to better understand the nature of relationships between alcohol availability, consumption and related harm.

The limited powers afforded to local authorities for controlling or managing alcohol availability in the UK mean it provides a useful example of the difficulties faced by practitioners in bringing evidence from availability research to bear at different stages in the policy process. These difficulties should be understood in the context of an evolving policy landscape. In the mid-20th century, the decline of temperance, the development of harm models focusing on individual problem drinkers and the increasing influence of deregulatory models of market governance led to moves away from a regulatory focus on outlet density [6]. Licensing was increasingly posited as an administrative process which should not seek to regulate availability to reduce consumption but, instead, limit itself to the protection of public order, amenity and public safety [7]. The Licensing Act (2003), around which current availability policy is structured, represented the apotheosis of this trend not only by removing statutory operating hours, but through its core principle that licensing authorities should principally act as mediators between stakeholders in a system primarily defined by market demand [8]. The consideration of ‘need’ for more alcohol outlets was explicitly disbarred. Outlet density considerations remained but only within limited local authority powers to consider using ‘cumulative impact policies’ to restrict the growth of groups of outlets in small geographic areas (e.g. a single street or square) [9]. The Scottish Licensing Act of 2005 adopted a similar approach but also required licensing boards to include a position on ‘overprovision’ in their Statements of Licensing Policy. Scotland was also the only UK jurisdiction to make the ‘protection of public health’ one of the specified licensing objectives on which licensing decisions must be based. For England and Wales, these objectives focused more narrowly on public safety, public order and protection of children.

The focus on individual drinkers and outlets meant translation of empirical evidence on the public health consequences of spatial and temporal availability was limited in these policy processes until the 1990s. Where public health concerns were present, licensing authorities in England and Wales could, in principle, rely on their own discretion in deciding whether an area had a ‘need’ for more alcohol outlets [10]. Legislation now requires cumulative impact and overprovision policies to be evidence based, pertain to specific outlets or local areas and make reference to the licensing objectives. Within this structure, local authorities often struggle to support their decision making with the available routine data which include police crime data, hospital emergency department statistics and licensing data on outlets but limited geographically linked information on longer-term harms. Health data are particularly problematic as it is typically only available at population-level and cannot demonstrate causal links between individual outlets or geographic areas and harmful outcomes [11]. A further complication has been the shift in retail from a predominantly on-sale market (where the spatial and temporal proximity of purchase, consumption and harm are well suited to observational study) to a market dominated by off sales (where purchase, consumption and harm are spatially and temporally disconnected). Although the UK context has unique features, these challenges are found in many other jurisdictions, and authorities seeking to use availability policies to tackle long-term public health harms are, therefore, in need of clear, robust evidence accounting for such challenges, applicable to the current policy context and identifying the relationship between measures of spatial and temporal availability and a range of both short- and long-term outcomes.

Previous reviews have examined the findings of the empirical evidence base [3],[4],[12]; therefore, we present here a critical review of the literature to identify its gaps and limitations and, focusing on the UK context as an example, assess its ability to speak to the above policy concerns. In particular, the paper aims to the following: (i) descriptively analyse the research design, setting and measures used in empirical analyses of availability; and (ii) highlight evidence gaps and limitations identified during the review and recommendations for future translational and non-translational research to address these.

## Methods

Two main datasets were used to assess the evidence base. The first was 59 studies included in a systematic review conducted in December 2008 of empirical spatial and temporal availability research, hereafter the Popova review [12]. The second was 79 studies included in a systematic review conducted by the authors to update the Popova review to January 2014 (see Appendix S1 for full reference list). Review methods mirrored Popova *et al.'s* and are reported in full in Appendix S1 alongside search results information. In brief, we searched Ovid Medline, Psycinfo, CiNAHL, Web of Knowledge and Embase using search terms relating to both spatial and temporal availability, alcohol consumption and alcohol-related harm. Studies were included in the final sample if they were English language, empirical primary studies conducted in Organisation for Economic Co-operation and Development countries between 2009 and January 2014, used measures of spatial availability or temporal availability as exposure measures, and quantified the association between these exposures and one or more alcohol-related outcome. Studies which did not objectively measure availability (e.g. perceived availability) were excluded as were before and after evaluation studies which measured impacts on outcome measures of a policy without measuring impacts on availability.

For each study, data on research design and setting, availability measures, and outcome measures were extracted. These data were used to define exactly which availability and outcome measures were being studied and to review study designs and analyses to identify research gaps and future priorities.

## Results

Of 138 studies reviewed, 32 from the Popova review did not directly or objectively measure availability. This includes evaluations of policy interventions where resultant changes in availability were not measured. The remaining 118 studies contained 136 measures of spatial availability and three of temporal availability (Table 1).

**Table 1:** Types of availability measure and outlet disaggregation in studies with outlet-level availability measures

Measure	Number of measures
**Spatial**	**136**
Outlet density	118
*Simple count*	*39*
*Weighted by area*	*20*
*Weighted by population*	*36*
*Weighted by roadway miles*	*22*
*Weighted by sales*	*1*
Proximity	16
*Euclidean*	*7*
*Travel distance*	*8*
*Cumulative distance*	*1*
Others	2
*Outlet clustering*	*1*
*Retail gravity*	*1*
**Temporal**	**3**
Late night outlet indicator	3
**Outlet disaggregation**	**170**
All outlets	40
Off-trade only	25
On-trade only	12
On-trade versus off-trade	48
Off-trade disaggregated (e.g. supermarkets vs. liquor stores)	9
On-trade disaggregated (e.g. bars vs. restaurants)	36

### Measures of spatial and temporal availability

Of the 136 spatial measures, 118 measured outlet density, 16 measured proximity and one each measured outlet clustering and ‘retail gravity’. Outlet density was generally measured as the number of outlets within a given area, and large or densely populated areas containing several thousand households were typically used (e.g. US zip codes or census tracts). Individual exposures are likely to vary markedly across these areas, creating the potential for ecological biases [13],[14]; although a small number of studies have used lower-level geographies (e.g. Cunradi *et al*. and Gorman *et al*. [15],[16]). Densities were often weighted by a denominator accounting for varying area characteristics, with denominators including the population, roadway miles or size of an area. The relative merits of these weighting options are unclear [17],[18], and some authors argue weighting leads to misconceptions as availability is experienced by individuals not populations [19]. For example, an area may have low outlet density if weighted by a large population, but individuals within that area still experience high availability. The main variations between proximity measures were whether Euclidean 20–22 or road [14],[23],[24] distances were used and whether distance to a single outlet [14],[21],[25] or cumulative distance to several outlets [26] was measured. The latter may be viewed as an alternative measure of outlet density; indeed, all proximity measures serve as proxies for outlet density to some extent and vice versa. The outlet clustering and retail gravity measures were unusual in their more sophisticated approach to measuring availability. The former used spatial cluster detection techniques to identify highly localised outlet hotspots rather than calculating outlet density across larger areas [27]. The retail gravity measure assumed drinkers were affected by all outlets but weighted effects by outlets' proximity and size in analyses [28].

For temporal availability, most studies evaluated policy changes affecting trading hours and provided no outlet-level measure. The only three outlet-level measures came from a single dataset identifying which Australian outlets had extended trading permits 29–31.

On-trade and off-trade outlet availability may have different impacts as may the different types of outlets within that dichotomy (e.g. bars vs. restaurants). Although only 40 availability measures grouped all outlets into a single category, just 36 disaggregated on-trade outlet types (typically hotels, restaurants and bars), and only nine disaggregated off-trade outlet types (supermarkets vs. liquor stores or government vs. private stores).

### Outcome measures

The outcome measures analysed for associations with availability in the empirical literature tend to focus on acute health and social outcomes and heavy alcohol consumption (Table 2). The 138 studies reviewed analysed 185 outcomes of which 122 addressed acute consumption or harms (particularly violence and driving-related harms), 25 addressed long-term consumption and just nine addressed chronic diseases or mortality. The remaining 29 outcomes included youth drinking, child abuse and social capital.

**Table 2:** Outcome measures in empirical availability studies

Outcome measure^a^	*n*
**Acute**	**122**
Consumption	16
*Binge drinking*	*16*
Harm	106
*Crime*	*9*
*Disorder*	*6*
*Driving-related*	*25*
*Acute harms to others*	*1*
*Injuries*	*7*
*Intimate partner violence*	*11*
*Emergency department admissions*	*3*
*Sexually transmitted infections*	*3*
*Suicide*	*3*
*Violence*	*38*
**Chronic**	**34**
Consumption	25
*Alcohol abuse*	*8*
*Average consumption*	*17*
Harm	9
*Chronic diseases or other morbidities*	*4*
*Mortality*	*5*
**Other****b**	**29**
Consumption	13
*Youth drinking*	*13*
Harm	11
*Child abuse*	*5*
*Other substance use*	*5*
*Youth deviance*	*1*
Other	5
*Social capital*	*2*
*Neighbourhood safety*	*2*
*No primary analysis*	*1*
**Total**	**185**

a Where studies analysed multiple measures within one low-level category (e.g. alcohol abuse, crime), the measure is only counted once to avoid skewing of counts by single studies using several similar measures.

b Outcomes are classed as ‘other’ where outcome could be either acute or chronic or where this is unclear.

### Research settings

The 138 studies were dominated by research from California (*n* = 35), the USA as a whole (*n* = 91) and, more recently, Australia (*n* = 20) (Table 3). Of the 106 studies which measured availability at the outlet level, just 13 were not from the USA or Australia, and only six were from Europe. Policy evaluation or natural experiment studies were more evenly distributed across developed nations, although the majority still came from Australia, Canada or the USA. However, the restricting of this review to English language papers is an important caveat to these figures.

**Table 3:** Study setting of empirical availability research

Location	All	Ecological studies	Policy evaluations and natural experiments	Studies directly measuring availability
Australia	20	14	6	17
Canada	10	2	8	4
Europe	12	6	6	6
Latin America	1	0	1	0
New Zealand	4	3	1	3
USA (California)	91 (35)	80 (31)	11 (4)	86 (35)
Total	138	105	33	116

In summary, the empirical evidence base demonstrates the impact of outlet density across large areas but offers less evidence on alternative or more localised forms of spatial availability or local variations in temporal availability. Evidence also tends to focus on a narrow range of aggregated outlet categories and research settings with a particular focus on acute outcome measures.

### Gaps and limitations in the empirical evidence base and its utility to UK licensing policy

Below, we present the major gaps and limitations identified during our review. In particular, we focus on challenges in translating existing research into policy using examples from the UK, although many of our points also have relevance to research goals beyond knowledge translation. The critiques are brought together in Table 4 which also contains our recommendations for addressing these points.

**Table 4:** Identified limitations in current availability research and recommended actions

Limitation	Recommendation(s)
1. There is a narrow focus in both theoretical and empirical literature on acute outcomes (e.g. crime, disorder and violence).	Research should examine the impact of availability on chronic health outcomes to develop an evidence base in this area Stakeholders should seek to maximise access to and utilisation of further spatial and temporal data on consumption to facilitate the above; potential approaches to achieving this include the following: (i) utilising existing consumption datasets where spatial data are collected but only available for analysis under special license systems; (ii) stakeholders lobbying for amendment of informed consent procedures for regularly collected data (e.g. annual national surveys) to allow access to spatial data; and (iii) researchers and funders seeking or allocating new monies to create spatial datasets.
2. Availability is often discussed or measured within highly aggregated outlet categories.	Where possible, researchers' analyses should stratify outlets within low-level categories (e.g. supermarkets, local pubs, nightclubs) to refine understandings of problematic forms of availability. Stakeholders should seek to maximise access to data to facilitate such analyses, for example, by (i) identifying and purchasing suitable market research data; (ii) lobbying governments to legislate to ensure relevant private data are available to researchers with appropriate safeguards to protect commercial interests; and (iii) conducting business surveys or crowdsourcing data, where the latter facilitates the public to voluntarily submit data on outlet characteristics (e.g. through advertising online submission forms) [32].
3. Weak linkage of theory, empirical analysis and policy practice	Researchers should analyse a broader range of availability measures to improve linkage between theory, empirical analysis and local licensing practice. Measures we view as priorities include those incorporating highly localised clustering of outlets, spatial patterning of temporal availability, dichotomous measures and composite indices. Low-level spatial data may be required to construct such measures. Analyses should explore whether relationships between availability and outcomes are discrete or continuous and linear or curvilinear to aid identification of target availability levels.
4. Empirical literature is largely drawn from the USA and Australia.	Analyses in more contexts, particularly European countries, are required to improve generalisability of the evidence base and to facilitate the development of a comparative availability literature.
5. Analyses do not recognise the spatial range of individuals' lives.	Researchers may attempt to use travel surveys or GPS data to generate maps of individuals' usual activities and link these to consumption and purchasing locations to understand the true spatiotemporal cost of purchase and consumption (e.g. whether the supermarket is next door to work or en route on the journey home) rather than simply measuring the distance between outlets and home. Even small-scale studies would be useful to inform large sample analyses
6. Subgroup level analyses are required.	Analyses should give greater consideration to the impact of availability on different population subgroups and drinking patterns of interest to policy makers (e.g. moderate drinkers vs. harmful drinkers) to aid decision making.
7. The interaction between price, place and availability is relatively unexplored.	Researchers should seek local and ideally outlet-level pricing, spending and price promotion data through business surveys, market research data or crowdsourcing (see limitation #2). This data should be incorporated into empirical analyses alongside area-level characterisations to aid unpicking of the price, place and availability interaction.
8. Online alcohol availability has not been considered in the literature to date.	New research should be undertaken examining the extent of online alcohol purchasing and the relationship of this to consumption behaviours and related harms to inform policy debate in this now well-established market sector.

#### A narrow focus

Studies have tended to focus on acute outcomes and heavy consumption with relatively little attention given to chronic disease, mortality and average consumption. In the UK, acute outcomes and heavy consumption are typically framed in licensing policy as problems of individual outlets or irresponsible drinkers. When seeking to reduce these acute harms, little attention is given in decision-making processes to concepts such as outlet density. Thus, the lack of evidence on longer-term health implications means public health practitioners have limited scope for making representations regarding controlling overall spatial and temporal availability within policy debate and licensing processes. For example, many Scottish licensing boards have reported that efforts to apply the protection of public health as a licensing objective are hampered by a lack of clear data [11], and this has also been identified as a key concern among licensing teams in England and Wales where public health became a statutory ‘Responsible Authority’ for local licensing in 2012 [33]. When evidence has been applied, licensing decisions in Scotland referencing public health concerns have sometimes been subjected to successful legal challenges around their failure to, in the words of one court ruling, ‘demonstrate a causal link between the particular mischief apprehended and the general terms of the policy itself’ [34]. The limitations of the available evidence were highlighted during the consultation on the 2012 Alcohol Strategy, when the UK Home Office stated that ‘local processes and data collection are insufficient’ to make its proposed introduction of a public health objective for cumulative impact policies in England and Wales practical [35]. Developing and disseminating data on the long-term health consequences of outlet density are, therefore, critical. This data should ideally include longitudinal spatially linked consumption data which can serve as markers for potential chronic harm.

#### Aggregated outlet categorisations

Current research generally considers outlets within highly aggregated categories (e.g. off-trade outlets, bars), meaning, there is a lack of clarity as to whether observed relationships apply to all outlet types. Attributing negative effects to broad outlet categories overlooks the wide diversity of outlets those categories contain, including sleepy pubs, crowded modern bars, supermarkets and 24-h booze stores. These distinctions feature in policy processes with Scottish licensing boards being encouraged to take account of different outlet types when developing their overprovision statements and, in England and Wales, a number of local authorities having sought to tailor cumulative impact policies to tackle ‘vertical drinking outlets’ while encouraging the establishment of food-led or family-oriented outlets (e.g. Newcastle City Council [36]).

Branas *et al*. have moved towards better outlet differentiation by controlling for sales figures in their analysis [28]; however, no similar studies were identified. Ideal data to amend this would detail outlet-level characteristics, such as licensing hours, capacity, sales data, clientele profile and other characteristics associated with harms [37]. Such datasets may be available from various commercial sources (e.g. market research agencies) or could be generated by researchers.

#### Weak linkage of theory, empirical analysis and policy practice

Availability measures sometimes appear poorly linked to prevailing availability theories which generally portray dense local clustering of outlets as providing the social conditions (e.g. in routine activities theory), social indicators (e.g. in social disorganisation theory) or clustering of problematic drinkers (e.g. in assortative drinking theories) which facilitate violence and other criminality via increased consumption or other mechanisms 38–40. Excepting the two more sophisticated measures above [27],[28], there has been little innovation in this area with the overwhelming majority of studies relying on generic measures [41]. Measures of outlet proximity are well matched to economically focused full-cost models as they proxy travel time and associated costs. However, it is less clear whether increased outlet density across large areas consistently indicates the social disorganisation which theorists argue increases perceived permissiveness of criminality 42–44 or the potential for crowding and violent collisions central to other theories [30]. This is particularly the case if outlets are evenly geographically distributed rather than in dense clusters. Socio-contextual data are used in some studies to supplement measures of outlet density and characterise levels of social disorganisation; however, innovation in measurement is required, and the localised outlet hotspots which concern theorists and policy practitioners may be better captured by approaches such as Grubesic and Pridemore's cluster detection measure [27].

Four further related problems were identified: (i) only a small number of studies have used direct measures of alcohol availability when evaluating the potential effectiveness of specific licensing approaches, such as lockout policies or discrete shifts in licensing laws 45–47. Instead, policy evaluations tend to use before and after research designs to evaluate changes in outcome measures without examining the degree of outlet-level change 47–52. In combination with the empirical focus on outlet density, this leads to the valid conclusion that reducing general availability is effective, but specific recommendations for strategies to manage local availability profiles or achieve desirable patterns of availability which policy makers can seek to implement within their own policy structures are lacking. (ii) Existing measures artificially separate temporal and spatial availability. No study combined these into a single measure (e.g. 24-h outlet proximity or late license bar density). (iii) Similarly, whether exposure to both high outlet density and high outlet proximity confers additive or multiplicative risks has not been explored, and investigation of composite indices of availability is required. (iv) Current measures do not address concerns regarding the presence, as opposed to number or proximity, of individual outlets or outlet types, such as living near a ‘bad bar’ or 24-h store or having an outlet within walking distance. This reflects an assumption that the relationships between availability and outcomes are continuous and linear rather than discrete or curvilinear. Few studies explore this assumption [24],[53],[54], but demonstrating non-linear relationships would facilitate understanding of target availability levels, harmful outlet types and the scale of change required to reduce harm in different contexts.

#### Limited research settings

Even acknowledging the linguistic limitations of our search, the relative lack of studies from outside the USA and Australia is problematic as other English-speaking nations have different outlet types and different norms, expectations, and values around them. For example, UK outlets are found in significant numbers wherever there are people. Almost all supermarkets and convenience stores sell alcohol and even small villages will typically have at least one pub which has traditionally been viewed as a hub of community life. Therefore, in important respects, the UK policy setting is different to the USA and Australia. UK policy makers may query the validity of empirical findings if they are explained by theory suggesting such commonplace outlets, even in large numbers, indicate social disorganisation. Some authors note that it is specific outlet types in specific areas, rather than outlets per se, which are problematic [16],[55], but this is not operationalised in the aggregated outlet categories of most empirical analyses. Research in a broader range of countries would improve the generalisability of findings, facilitate cross-national comparative analyses and enhance policy makers' ability to provide plausible policy rationales in more diverse policy contexts.

#### Spatial range of individuals' lives

Recent evidence suggests alcohol availability within an individual's residential area is not correlated with their actual exposure to alcohol outlets due to their additional exposure while outside the home [56]. In the UK and many other countries, this issue is complicated by recent increases in online sales with home delivery and the dominance of the alcohol market by major supermarkets to which people travel for a large shop. The different spatial ranges of individual's daily lives make local availability a poor proxy for outlet exposure and imply existing literature risks misattribution of harmful impacts to drinkers' local outlets. Drawing on travel surveys or utilising GPS technology within mobile devices presents new opportunities for mapping consumer behaviour which may be useful here (akin to Basta *et al*. [56]).

#### Subgroup analyses

Research designs appear poorly linked with the specifics of policy concerns, such as the need to avoid penalising the ‘responsible majority’, an argument deployed extensively by UK industry lobbyists when challenging legislation on both price and availability [57]. Furthermore, with the exception of young people (e.g. Chen *et al*., Reboussin *et al*., Chilenski *et al*. 58–60) and US ethnic groups (e.g. Truong and Sturm, Nielsen *et al*., Tobler *et al*. 61–63), the impacts of availability on population subgroups of interest are rarely explored. Similarly, analysis of multiple consumption dimensions, such as frequency, average and heavy episodic consumption, which feature heavily in policy debate and drinking guidelines, is rarely a focus of study 64–66. For research to provide maximum utility to policy makers, demonstrating that interventions are well targeted and complement existing policy is crucial.

#### Interaction of price, place and availability

Little research to date has explored the relationships between availability, price and place 67–70. Increased price competition is a product of high availability contexts [71], and this may partially account for the relationship between outlet density and harm. Availability effects may also be moderated by pricing in lower-income areas where drinkers, on average, purchase cheaper alcohol [72] often in larger containers facilitating heavier consumption. For example, a recent UK study found that independent retailers in deprived neighbourhoods were more likely to stock cheap, strong products as supermarkets were better geared to cut prices on premium brands [73]. Understanding these processes is important as pricing regulation may be an effective policy response to increased availability and again suggests disaggregation of outlets may be important. Local price indices may be useful inputs to analyses, but these are typically unavailable at sufficiently disaggregated geographies. Such indices also indicate the range of prices customers face, which are unrepresentative of the prices they choose to pay. Ideally, outlet-level pricing and purchasing data would be used alongside other outlet characteristics, such as prices for popular brands or outlet price distributions.

#### Online availability

The role of the Internet in changing alcohol availability has received little attention. Online retailers deliver alcohol as part of weekly grocery shopping or convenience purchases, provide access to bulk or specialised product purchases, and supply or restock parties. In all instances, availability has increased beyond the detection of the spatial maps used in most analyses, and to date, it is unclear what the extent or focus of policy concern around internet sales should be. New data detailing online purchasing behaviours are emerging from the International Alcohol Control Study [74], and spending surveys may provide an alternative data source which could be linked, using statistical models, to consumption data to estimate the proportion of purchases which are made online.

## Discussion

The overarching finding of this review is that although a large body of research exists on the impacts of spatial and temporal alcohol availability, this evidence base is somewhat generic and high level in terms of the research settings, the availability and outcome measures used, and the conclusions individual studies can offer. Although recommendations to control alcohol availability remain valid, specific guidance on the forms of availability which should be addressed, the effective controls which should be applied or the full range of intervention outcomes which may be expected is difficult to derive. This lack of specificity has concrete policy implications in countries such as the UK where, for example, legal appeals against licensing restrictions often hinge on the validity of evidence linking density to harm.

This review has several imitations. First, it excluded non-English language studies for practical reasons, and this may have excluded relevant literature from European countries in particular. Second, the primary focus was on studies measuring alcohol availability, and in the updated systematic review, this excluded literature evaluating policy interventions without directly measuring resultant changes in availability. Third, we have not sought to systematically analyse whether different availability measures are consistently associated with particular outcomes. As with other reviews to date, we have found such analyses are hindered by substantial heterogeneity in data quality, analytical techniques and outcome measures. On this topic, Livingston *et al*. have noted similar studies often yield contradictory findings [41], such as bar density but not liquor store density being predictive of violence in one study (e.g. Cunradi *et al*. [15]), but the exact opposite (i.e. liquor stores being predictive but bars not) being true in another (e.g. Cunradi *et al*. [75]), and this supports our general recommendation for more disaggregated analyses to unpick these contradictions. Finally, we have not critiqued the statistical methods used in the reviewed studies, although others have done so within the broader ‘neighbourhood effects’ literature [76]. While not the focus of this paper, such critiques are valid, applicable to the alcohol availability evidence base and offer useful guidance for developing research methods in this area.

The UK alcohol licensing system has provided an example context in this review for discussing questions of translation of research into practice. The limited scope provided by this system for the strategic control of alcohol availability centres on the facility for English licensing authorities to develop cumulative impact policies and the requirement for Scottish licensing boards to provide statements on over-provision and consider public health within licensing decisions. In both countries, public health- or social order-oriented decisions have been challenged in the courts by highly specialist licensing lawyers leading to other authorities abandoning plans under threat of challenge [77]. Innovative data-linking approaches, such as the ‘Cardiff Model’, have allowed acute health issues (specifically A&E admissions) to play a role in licensing in some areas; however, in both England and Scotland, the involvement of health authorities in the licensing process has been hampered by a lack of relevant evidence, difficulties in translating high-level empirical research to local contexts and legal challenges to the validity of data during appeal processes. Translating research on alcohol into policy is difficult, especially in a policy environments characterised by procedural, discretionary, pragmatic and legalistic practices which demand that evidence has demonstrable relevance to local specificities. However, such environments are commonplace around the world, and if the research community aspires to informing evidence-based policy, it is imperative for research to be designed so that its findings can be operationalised in such contexts. However, this is not a one-sided proscription; our consideration of the UK example also highlights a burden on policy actors to communicate their evidence needs effectively to researchers and for policy makers to consider how policy processes can be revised to create a role for research evidence which, unless specifically commissioned, rarely emerges in precisely the desired format.

Our recommendation for greater analytic innovation should not be interpreted as suggesting research has stood still. Availability analyses have developed to explore, for example, the relative effects of environmental and individual factors on outcomes [14],[22], the relationship between social disorganisation, availability and harm [43], availability's impacts on social capital [17],[78],[79], and new spatial analysis techniques. Moreover, although our focus has been on research of direct relevance to jurisdictions such as the UK which operating local licensing policies, there is a substantial body of relevant evidence pertaining to changes within state alcohol monopolies from which important conclusions can be drawn [1],[80]. Nonetheless, the policy context outlined above means our over-riding message for researchers, irrespective of the translational motivations of their work, is for this innovation to be applied to further areas, including empirical research design, construction of measures and use of data. Table 4 presents our recommendations to achieve these research goals, and it particularly highlights the need for (i) sourcing and utilising new datasets and (ii) developing research designs and analyses which focus on identifying the range of specific forms of availability which require controls and which outcomes, population groups and contexts may be affected by these. We encourage local licensing stakeholders and researchers alike to engage with and develop these recommendations to allow evidence to play a greater role in alcohol licensing policy.

## Conclusions

Current evidence supports conclusions that controlling the spatial and temporal availability of alcohol is a key intervention for reducing alcohol consumption and related harms. However, this review has identified a series of methodological gaps and limitations in the existing evidence base. These hinder the translation of research into evidence-based policy recommendations. Research stakeholders should focus resources on resolving data needs and better integration of theory, empirical analysis and policy practice to facilitate a step change in availability research.

## Supporting Information

Appendix S1 Updating the Popova review to January 2014
