# Supplementary material for: The impact of spatial and temporal availability of alcohol on its consumption and related harms: A critical review in the context of UK licensing policies
Source: Drug Alcohol Rev. 2014 Sep 4;33(5):515–25. doi: 10.1111/dar.12191 (PMC4313683; doi:10.1111/dar.12191)
Supplement: Appendix S1 — Updating the Popova review to January 2014 [file dar0033-0515-sd1.docx]

**ONLINE APPENDIX**

**Updating the Popova review to January 2014**

Popova et al. conducted a systematic review of the relationship between spatial or temporal availability and a range of outcomes and searched for studies published between January 2000 and December 2008. This search yielded 59 studies (44 spatial and 15 temporal). To update this systematic review to January 2014 we largely followed Popova et al.’s methodology and report on this below. The main difference with the Popova review was that as we were only interested in studies which measured availability at the outlet level and not in studies which examined the effect of policy changes without directly measuring changes in availability, we excluded the latter type of study.

*Databases and search terms*

Databases searched were Ovid Medline, Psycinfo, CiNAHL, Web of Knowledge and Embase. Combinations of the following terms were used to search these databases:

“physical availability” , “alcohol* availability” , “alcohol outlet*” , “liquor outlet*” , “outlet densit*”, ”“hours of sale*” , “alcohol sale*” , “trading hours”, “alcohol* drink*” , “alcohol* consumption”, “alcohol* use”, drink*, binge*, drunk*, intoxicat*, “blood alcohol content”, morbidity, mortality, fatality, disease*, accident*, wound*, injur*, admission*, harm*, death*, driving, casualt*, “motor-vehicle casualt*”, “motor-vehicle collision*”, “motor-vehicle accident*”, “road crash*”, “road traffic accident*”, DUI, “driving under the influence”, crime or criminal*, violen*, harm*, damage*, assault*, trauma*, fight*, problem*, disorder, murder*, homicide*.

*Inclusion and exclusion criteria*

Studies were included in the final sample if:

- They were empirical primary studies conducted in OECD countries and published between 2009 and April 2014;
- Spatial availability (defined as the number of outlets) or temporal availability (defined as the hours or days of sale) of alcohol was the exposure measure;
- The association between spatial and temporal availability and one or more alcohol-related outcome was quantified;
- They were English language publications.

Studies were excluded if:

- They were systematic reviews or meta-analyses;
- They contained no empirical analysis of the relationship between spatial or temporal alcohol availability and an alcohol-related outcome;
- Availability exposure was not objectively measured (e.g. studies relying on subjective perceptions of availability or before and after policy evaluations with no availability measure were excluded);
- They duplicated results presented in another publication.

*Results*

After duplicates were removed, 712 articles were screened for inclusion and, of these, 115 were selected for detailed reading. A final total of 79 studies were found to meet the criteria for inclusion (Figure A1).

*Figure A1: PRISMA diagram of update of Popova review to 2014*

After duplicates removed: Total: 712

79 articles included for data extraction

36 excluded on further reading

597 excluded on title or abstract

115 full articles retrieved

**Example search strategy: Ovid Medline (search undertaken January 2014)**

| 1. ("physical availability" or "availability of alcohol" or "alcohol* availability" or "alcohol* outlet*" or "liquor outlet*" or "outlet densit*" or "outlet concentration" or "licens* outlet*" or "licens* premise*").ab. or ("physical availability" or "availability of alcohol" or "alcohol* availability" or "alcohol* outlet*" or "liquor outlet*" or "outlet densit*" or "outlet concentration" or "licens* outlet*" or "licens* premise*").ti. |
| --- |
| 1. ("hours of sale*" or "alcohol* sale*" or "trading hours" or "drinking hours" or "licens* hours" or "hours of trad*").ab. or ("hours of sale*" or "alcohol* sale*" or "trading hours" or "drinking hours" or "licens* hours" or "hours of trad*").ti. |
| 1. exp Alcohol Drinking/ or exp Alcoholic Beverages/ or exp Alcoholic Intoxication/ |
| 1. ("alcohol* consumption" or "alcohol* use" or drink* or binge* or drunk* or intoxicat* or "blood alcohol content").ab. or ("alcohol* consumption" or "alcohol* use" or drink* or binge* or drunk* or intoxicat* or "blood alcohol content").ti. |
| 1. exp morbidity/ or exp mortality/ |
| 1. exp Accidents, Traffic/ or exp "Wounds and Injuries"/ |
| 1. exp Disease/ |
| 1. exp Accidents/ |
| 1. exp Hospitalization/ |
| 1. exp Death/ |
| 1. (morbidit* or mortalit* or fatalit* or disease* or accident* or wound* or injur* or admission* or hospitalization* or harm* or death*).ab. or (morbidit* or mortalit* or fatalit* or disease* or accident* or wound* or injur* or admission* or hospitalization* or harm* or death*).ti. |
| 1. Automobile Driving/ |
| 1. (driving or casualt* or "motor-vehicle casualt*" or "motor-vehicle collision*" or "motor-vehicle accident*" or "road crash*" or "road traffic accident*" or DUI or "driving under the influence").ab. or (driving or casualt* or "motor-vehicle casualt*" or "motor-vehicle collision*" or "motor-vehicle accident*" or "road crash*" or "road traffic accident*" or DUI or "driving under the influence").ti. |
| 1. exp Crime/ |
| 1. (crime or criminal* or violen* or damage* or assault* or trauma* or fight* or problem* or disorder* or murder* or homicide*).ab. or (crime or criminal* or violen* or damage* or assault* or trauma* or fight* or problem* or disorder* or murder* or homicide*).ti. |
| 1. 1 or 2 |
| 1. 3 or 4 |
| 1. 5 or 6 or 7 or 8 or 9 or 10 or 11 |
| 1. 12 or 13 |
| 1. 14 or 15 |
| 1. 17 or 18 or 19 or 20 |
| 1. 16 and 21 |
| 1. limit 22 to (english language and yr="2009 - 2012") |

**References**

[1] Ahern J, Margerison-Ziko C, Hubbard A, Galea S. Alcohol outlets and binge drinking in urban neighborhoods: The implications of nonlinearity for intervention and policy. Am J Public Health 2013;103**:**e81-e87

[2] Branas CC, Elliot MR, Richmond TS, Culhane DP, Wiebe DJ. Alcohol Consumption, Alcohol Outlets and the Risk of Being Assaulted With a Gun. Alcohol Clin Exp Res 2009;33**:**1-10

[3] Breen C, Shakeshaft A, Slade T, et al. Do Community Characteristics Predict Alcohol-Related Crime? Alcohol Alcoholism 2011;46**:**464-70

[4] Briscoe S, Donnelly N. Problematic licensed premises for assault in inner Sydney, Newcastle and Wollongong. Australian and New Zealand Journal of Criminology 2003;36**:**18-33

[5] Britt HR, Carlin BP, Toomey TL, Wagenaar AC. Neighborhood level spatial analysis of the relationship between alcohol outlet density and criminal violence. Environ Ecol Stat 2005;12**:**411-26

[6] Cameron MP, Cochrane W, McNeil K, et al. Alcohol outlet density is related to police events and motor vehicle accidents in Manukau City, New Zealand. Aust N Z J Public Health 2012;36**:**537-42

[7] Carpenter CS, Eisenberg D. Effects of Sunday Sales Restrictions on Overall and Day-Specific Alcohol Consumption: Evidence From Canada. J Stud Alcohol Drugs 2009;70**:**126-33

[8] Chen M, Grube JW, Gruenewald PJ. Community alcohol outlet density and underage drinking. Addiction 2010;105**:**270-8

[9] Chen MJ, Gruenewald PJ, Remer LG. Does Alcohol Outlet Density Affect Youth Access to Alcohol? J Adolesc Health 2009;44**:**582-9

[10] Chikritzhs T, Stockwell T. The impact of later trading hours for Australian public houses (hotels) on levels of violence. J Stud Alcohol 2002;63**:**591-9

[11] Chikritzhs T, Stockwell T. The impact of later trading hours for hotels on levels of impaired driver road crashes and driver breath alcohol levels. Addiction 2006;101**:**1254-64

[12] Chikritzhs T, Stockwell T. The impact of later trading hours for hotels (public houses) on breath alcohol levels of apprehended impaired drivers. Addiction 2007;102**:**1609-17

[13] Chilenski SM, Greenberg MT, Feinberg ME. The Community Substance Use Environment: The Development and Predictive Ability of a Multi-method and Multiple-reporter Measure. J Community Appl Soc 2010;20**:**57-71

[14] Cohen DA, Ghosh-Dastidar B, Scribner R, et al. Alcohol outlets, gonorrhea, and the Los Angeles civil unrest: a longitudinal analysis. Soc Sci Med 2006;62**:**3062-71

[15] Cooper HL, Bonney LE, Ross Z, et al. The aftermath of public housing relocation: Relationship to substance misuse. Drug Alcohol Depen 2013;133**:**37-44

[16] Cunradi C, Mair C, Ponicki W, Remer L. Alcohol Outlet Density and Intimate Partner Violence-Related Emergency Department Visits. Alcohol Clin Exp Res 2012;35**:**847-53

[17] Cunradi CB, Mair C, Ponicki W, Remer L. Alcohol outlets, neighborhood characteristics, and intimate partner violence: ecological analysis of a California city. J Urban Health 2011;88**:**191-200

[18] Day P, Breetzke G, Kingham S, Campbell M. Close proximity to alcohol outlets is associated with increased serious violent crime in New Zealand. Aust N Z J Public Health 2012;36**:**48-54

[19] Donnelly N, Poynton S, Weatherburn D, Bamford E, Nottage J. Liquor outlet concentrations and alcohol-related neighbourhood problems. Alcohol Studies Bulletin 2006;8**:**1-16

[20] Duailibi S, Ponicki W, Grube J, et al. The effect of restricting opening hours on alcohol-related violence. Am J Public Health 2007;97**:**2276-80

[21] Escobedo LG, Ortiz M. The relationship between liquor outlet density and injury and violence in New Mexico. Accident Anal Prev 2002;34**:**689-94

[22] Forsyth AJ, Davidson N. Community off-sales provision and the presence of alcohol-related detritus in residential neighbourhoods. Health Place 2010;16**:**349-58

[23] Freisthler B, Gruenewald PJ. Where the individual meets the ecological: A study of parent drinking patterns, alcohol outlets and child physical abuse. Alcohol Clin Exp Res 2013;37**:**993-1000

[24] Freisthler B, Gruenewald PJ, Remer LG, Lery B, Needell B. Exploring the spatial dynamics of alcohol outlets and Child Protective Services referrals, substantiations, and foster care entries. Child Maltreatment 2007;12**:**114-24

[25] Freisthler B, Midanik LT, Gruenewald PJ. Alcohol outlets and child physical abuse and neglect: applying routine activities theory to the study of child maltreatment. J Stud Alcohol 2004;65**:**586-92

[26] Freisthler B, Needell B, Gruenewald PJ. Is the physical availability of alcohol and illicit drugs related to neighborhood rates of child maltreatment? Child Abuse Neglect 2005;29**:**1049-60

[27] Freisthler B, Weiss RE. Using Bayesian space-time models to understand the substance use environment and risk for being referred to child protective services. Subst Use Misuse 2008;43**:**239-51

[28] Freisthler B, Byrnes HF, Gruenewald PJ. Alcohol outlet density, parental monitoring, and adolescent deviance: A multilevel analysis. Child Youth Serv Rev 2009;31**:**325-30

[29] Gorman DM, Speer PW, Gruenewald PJ, Labouvie EW. Spatial dynamics of alcohol availability, neighborhood structure and violent crime. J Stud Alcohol 2001;62**:**628-36

[30] Gorman DM, Zhu L, Horel S. Drug 'hot-spots', alcohol availability and violence. Drug Alcohol Rev 2005;24**:**507-13

[31] Grubesic TH, Pridemore WA, Williams DA, Philip-Tabb L. Alcohol outlet density and violence: The role of risky retailers and alcohol-related expenditures. Alcohol Alcoholism 2013;48**:**613-9

[32] Grubesic TH, Pridemore WA. Alcohol outlets and clusters of violence. Int J Health Geogr 2011;10**:**

[33] Gruenewald PJ, Freisthler B, Remer L, LaScala EA, Treno A. Ecological models of alcohol outlets and violent assaults: crime potentials and geospatial analysis. Addiction 2006;101**:**666-77

[34] Gruenewald PJ, Freisthler B, Remer L, et al. Ecological associations of alcohol outlets with underage and young adult injuries. Alcohol Clin Exp Res 2010;34**:**519-27

[35] Gruenewald PJ, Johnson FW, Ponicki WR, Remer LG, LaScala EA. Assessing correlates of the growth and extent of methamphetamine abuse and dependence in California. Subst Use Misuse 2010;45**:**1948-70

[36] Gruenewald PJ, Johnson FW, Treno AJ. Outlets, drinking and driving: a multilevel analysis of availability. J Stud Alcohol 2002;63**:**460-8

[37] Gruenewald PJ, Remer L. Changes in outlet densities affect violence rates. Alcohol Clin Exp Res 2006;30**:**1184-93

[38] Gruenewald PJ, Johnson FW. Drinking, Driving, and Crashing: A Traffic-Flow Model of Alcohol-Related Motor Vehicle Accidents. J Stud Alcohol Drugs 2010;71**:**237-48

[39] Gyimah-Brempong K. Alcohol availability and crime: Evidence from census tract data. South Econ J 2001;68**:**2-21

[40] Halonen JI, Kivimäkim M, Virtanen M, et al. Living in proximity of a bar and risky alcohol behaviours: A longitudinal study. Addiction 2012;108**:**320-8

[41] Halonen JI, Kivimäkim M, Virtanen M, et al. Proximity of off-premise alcohol outlets and alcohol consumption: A cohort study. Drug Alcohol Depen 2014;132**:**295-300

[42] Hough M, Hunter G. The 2003 Licensing Act's impact on crime and disorder: An evaluation. Criminology & Criminal Justice 2008;8**:**239-60

[43] Iritrani BJ, Waller MW, Tucker Halpern C, et al. Alcohol outlet density and young women's perpetration of violence toward male intimate partners. Journal of Family Violence 2013;28**:**459-70

[44] Johnson FW, Gruenewald PJ, Remer LG. Suicide and alcohol: Do outlets play a role? Alcohol Clin Exp Res 2009;2124-33

[45] Kavanagh AM, Kelly MT, Krnjacki L, et al. Access to alcohol outlets and harmful alcohol consumption: a multi-level study in Melbourne, Australia. Addiction 2011;106**:**1772-9

[46] Kuntsche EN, Kuendig H. Do school surroundings matter? Alcohol outlet density, perception of adolescent drinking in public, and adolescent alcohol use. Addict Behav 2005;30**:**151-8

[47] Kypri K, Bell ML, Hay GC, Baxter J. Alcohol outlet density and university student drinking: a national study. Addiction 2008;103**:**1131-8

[48] Kypri K, Jones C, McElduff P, Barker D. Effects of restricting pub closing times on night-time assaults in an Australian city. Addiction 2011;106**:**303-10

[49] Lange JE, Voas RB. Youth escaping limits on drinking: binging in Mexico. Addiction 2000;95**:**521-8

[50] Lapham SC, Gruenwald PJ, Remer L, Layne L. New Mexico's 1998 drive-up liquor window closure. Study I: effect on alcohol-involved crashes. Addiction 2004;99**:**598-606

[51] LaScala EA, Johnson FW, Gruenewald PJ. Neighborhood characteristics of alcohol-related pedestrian injury collisions: a geostatistical analysis. Prevention Science 2001;2**:**123-34

[52] Liang W, Chikritzhs T. Revealing the link between licensed outlets and violence: Counting venues versus measuring alcohol availability. Drug Alcohol Rev 2012;30**:**524-35

[53] Lipton R, Gruenewald P. The spatial dynamics of violence and alcohol outlets. J Stud Alcohol 2002;63**:**187-95

[54] Lipton R, Yang X, Braga AA, et al. The geography of violence, alcohol outlets and drug arrests in Boston. Am J Public Health 2013;103**:**657-64

[55] Livingston M. A longitudinal analysis of alcohol outlet density and assault. Alcohol Clin Exp Res 2008;32**:**1074-9

[56] Livingston M. Alcohol outlet density and assault: a spatial analysis. Addiction 2008;103**:**619-28

[57] Livingston M. The ecology of domestic violence: The role of alcohol outlet density. Geospatial Health 2010;5**:**November

[58] Livingston M. A longitudinal analysis of alcohol outlet density and domestic violence. Addiction 2011;106**:**May-919

[59] Livingston M. Alcohol outlet density and harm: Comparing the impacts on violence and chronic harms. Drug Alcohol Rev 2011;30**:**515-23

[60] Livingston M, Laslett AM, Dietze P. Individual and community correlates of young people's high-risk drinking in Victoria, Australia. Drug Alcohol Depen 2008;98**:**241-8

[61] Lo CC, Weber J, Cheng TC. A spatial analysis of student binge drinking, alcohol-outlet density and social disadvantages. Am J Addiction 2013;22**:**401

[62] Lo CC, Weber J, Cheng TC. Urban-rural differentials: A spatial analysis of Alabama students' recent alcohol use and marijuana use. Am J Addiction 2014;22**:**188-96

[63] Lovenheim MF, Steefel DP. Do Blue Laws Save Lives? The Effect of Sunday Alcohol Sales Bans on Fatal Vehicle Accidents. J Policy Anal Manage 2011;30**:**798-820

[64] Mair C, Gruenewald PJ, Ponicki WR, Remer L. Varying impacts of alcohol outlet densities on violent assaults: Explaining differences across neighborhoods. J Stud Alcohol Drugs 2013;74**:**50-8

[65] Maloney MT, Rudbeck JC. The outcome from legalizing Sunday packaged alcohol sales on traffic accidents in New Mexico. Accid Anal Prev 2009;41**:**1094-8

[66] Mazerolle L, White G, Ransley J, Ferguson P. Violence in and around Entertainment Districts: A Longitudinal Analysis of the Impact of Late-Night Lockout Legislation. Law & Policy 2012;34**:**55-79

[67] McKinney CM, Caetano R, Harris TR, Ebama MS. Alcohol availability and intimate partner violence among US couples. Alcohol Clin Exp Res 2009;33**:**January

[68] McKinney CM, Chartier KG, Caetano R, Robert Harris T. Alcohol availability and neighborhood poverty and their relationship to binge drinking and related problems among drinkers in committed relationships. J Interpers Violence 2012;27**:**2703-27

[69] McMillan GP, Hanson TE, Lapham SC. Geographic variability in alcohol-related crashes in response to legalized Sunday packaged alcohol sales in New Mexico. Accident Anal Prev 2007;39**:**252-7

[70] McMillan GP, Lapham S. Effectiveness of bans and laws in reducing traffic deaths: legalized Sunday packaged alcohol sales and alcohol-related traffic crashes and crash fatalities in New Mexico. Am J Public Health 2006;96**:**1944-8

[71] Milam AJ, Furr-Holden CDM, Cooley-Strickland MC, Bradshaw CP, Leaf PJ. Risk for exposure to alcohol, tobacco and other drugs on the route to and from school: The role of alcohol outlets. Prevention Science 2014;15**:**12-21

[72] Newton A, Sarker SJ, Pahal GS, van den Bergh E, Young C. Impact of the new UK licensing law on emergency hospital attendances: a cohort study. Emerg Med J 2007;24**:**532-4

[73] Nielsen AL, Martinez R, Lee MT. Alcohol, ethnicity, and violence: The role of alcohol availability for Latino and black aggravated assaults and robberies. Sociol Quart 2005;46**:**479-502

[74] Norstrom T. Outlet density and criminal violence in Norway, 1960-1995. J Stud Alcohol 2000;61**:**907-11

[75] Norstrom T, Skog OJ. Saturday opening of alcohol retail shops in Sweden: an experiment in two phases. Addiction 2005;100**:**767-76

[76] Palk GRM, Davey JD, Freeman JE. The impact of a lockout policy on levels of alcohol-related incidents in and around licensed premises. Police Practice & Research: An International Journal 2010;11**:**5-15

[77] Pasch KE, Hearst MO, Nelson MC, Forsyth A, Lytle LA. Alcohol outlets and youth alcohol use: Exposure in suburban areas. Health Place 2009;15**:**642-6

[78] Pereira G, Wood L, Foster S, Haggar F. Access to alcohol outlets, alcohol consumption and mental health. PLoS One 2013;8**:**e53461

[79] Peterson RD, Krivo LJ, Harris MA. Disadvantage and neighborhood violent crime: Do local institutions matter? J Res Crime Delinq 2000;37**:**31-63

[80] Picone G, MacDougald J, Sloan F, Platt A, Kertesz S. The effects of residential proximity to bars on alcohol consumption. Int J Health Care Finance Econ 2010;10**:**347-67

[81] Pollack CE, Cubbin C, Ahn D, Winkleby M. Neighbourhood deprivation and alcohol consumption: does the availability of alcohol play a role? Int J Epidemiol 2005;34**:**772-80

[82] Ponicki WR, Gruenewald PJ, Remer LG. Spatial panel analyses of alcohol outlets and motor vehicle crashes in California: 1999-2008. Accid Anal Prev 2013;55**:**135-43

[83] Pridemore WA, Grubesic TH. Community organization moderates the effect of alcohol outlet density on violence. Br J Sociol 2012;63**:**680-703

[84] Pridemore WA, Grubesic TH. Alcohol outlets and community levels of interpersonal violence: Spatial density, outlet type and seriousness of assault. J Res Crime Delinq 2013;50**:**132-59

[85] Ragnarsdóttir P, Kjartansdóttir A, Davíðsdóttir S. Effects of extended alcohol serving-hours in Reykjavik. In: Room R, eds. The Effects of Nordic Alcohol Policies: What happens to drinking and harm when alcohol controls change?Helsinki: NAD Publication No. 42, 2002:86-92.

[86] Reboussin BA, Song EY, Wolfson M. The Impact of Alcohol Outlet Density on the Geographic Clustering of Underage Drinking Behaviors within Census Tracts. Alcohol Clin Exp Res 2011;35**:**1541-9

[87] Reid RJ, Hughey J, Peterson NA. Generalizing the alcohol outlet-assaultive violence link: evidence from a U.S. midwestern city. Subst Use Misuse 2003;38**:**1971-82

[88] Resko SM, Walton MA, Bingham C, et al. Alcohol Availability and Violence among Inner-City Adolescents: A Multi-Level Analysis of the Role of Alcohol Outlet Density. Am J Community Psychol 2010;46**:**253-62

[89] Roman CG, Reid SE. Assessing the relationship between alcohol outlets and domestic violence: Routine activities and the neighborhood environment. Violence Vict 2012;27**:**811-28

[90] Rossow I, Norström T. The impact of small changes in bar closing hours on violence. The Norwegian experience from 18 cities. Addiction 2012;107**:**530-7

[91] Rowland B, Toumbourou JW, Satyen L, et al. Associations between alcohol outlet densities and adolescent alcohol consumption: A study in Australian students. Addict Behav 2014;39**:**282-8

[92] Schofield TP, Denson T.F. Alcohol outlet business hours and violent crime in New York State. Alcohol Alcoholism 2013;48**:**363-9

[93] Schofield TP, Denson T.F. Temporal alcohol availability predicts first-time drunk driving but not repeat offending. PLoS One 2013;8**:**e71169

[94] Schonlau M, Scribner R, Farley TA, et al. Alcohol outlet density and alcohol consumption in Los Angeles county and southern Louisiana. Geospatial Health 2008;3**:**91-101

[95] Schootman M, Deshpande AD, Lynskey MT, et al. Alcohol outlet availability and excessive alcohol consumption in breast cancer survivors. Journal of Primary Care and Community Health 2012;4**:**50-8

[96] Scribner R, Mason K, Theall K, et al. The contextual role of alcohol outlet density in college drinking. J Stud Alcohol Drugs 2008;69**:**112-20

[97] Scribner RA, Cohen DA, Fisher W. Evidence of a structural effect for alcohol outlet density: a multilevel analysis. Alcohol Clin Exp Res 2000;24**:**188-95

[98] Scribner R, Theall KP, Ghosh-Dastidar B, et al. Determinants of social capital indicators at the neighborhood level: A longitudinal analysis of loss of off-sale alcohol outlets and voting. J Stud Alcohol Drugs 2007;68**:**934-43

[99] Sebert Kuhlmann AK, Brett J, Thomas D, Sain SR. Environmental characteristics associated with pedestrian-motor vehicle collisions in Denver, Colorado. Am J Public Health 2009;99**:**1632-7

[100] Shimotsu ST, Jone-Webb RJ, MacLehose RF, et al. Neighborhood socioeconomic characteristics, the retail environment and alcohol consumption: A multilevel analysis. Drug Alcohol Depen 2013;132**:**449-56

[101] Spoerri A, Zwahlen M, Panczak R, Egger M, Huss A. Alcohol-selling outlets and mortality in Switzerland: the Swiss National Cohort. Addiction 2013;108**:**1603-11

[102] Stanley LR, Henry KL, Swaim RC. Physical, Social, and Perceived Availabilities of Alcohol and Last Month Alcohol Use in Rural and Small Urban Communities. J Youth Adolescence 2011;40**:**1203-14

[103] Stockwell T, Zhao J, Macdonald S, et al. Changes in per capita alcohol sales during the partial privatization of British Columbia's retail alcohol monopoly 2003-2008: A multi-level local area analysis. Addiction 2009;104**:**November

[104] Stockwell T, Zhao J, Macdonald S, et al. Impact on alcohol-related mortality of a rapid rise in the density of private liquor outlets in British Columbia: a local area multi-level analysis. Addiction 2011;106**:**768-76

[105] Stockwell T, Zhao J, Martin G, et al. Minimum Alcohol Prices and Outlet Densities in British Columbia, Canada: Estimated Impacts on Alcohol-Attributable Hospital Admissions. Am J Public Health 2013;103**:**2014-20

[106] Theall KP, Scribner R, Cohen D, et al. Social capital and the neighborhood alcohol environment. Health Place 2009;15**:**323-32

[107] Theall KP, Scribner R, Cohen D, et al. The Neighborhood Alcohol Environment and Alcohol-Related Morbidity. Alcohol Alcoholism 2009;44**:**491-9

[108] Theall KP, Lancaster BP, Lynch S, et al. The Neighborhood Alcohol Environment and At-Risk Drinking Among African-Americans. Alcohol Clin Exp Res 2011;35**:**996-1003

[109] Tobler AL, Komro KA, Maldonado-Molina MM. Relationship Between Neighborhood Context, Family Management Practices and Alcohol Use Among Urban, Multi-ethnic, Young Adolescents. Prevention Science 2009;10**:**313-24

[110] Toomey TL, Erickson DJ, Bradley PC, et al. The association between density of alcohol establishments and violent crime within urban neighborhoods. Alcohol Clin Exp Res 2012;36**:**1468-73

[111] Toomey TL, Erickson DJ, Carlin BP, et al. Is the Density of Alcohol Establishments Related to Nonviolent Crime? J Stud Alcohol Drugs 2012;73**:**21-5

[112] Treno AJ, Grube JW, Martin SE. Alcohol availability as a predictor of youth drinking and driving: a hierarchical analysis of survey and archival data. Alcohol Clin Exp Res 2003;27**:**835-40

[113] Treno AJ, Gruenewald PJ, Johnson FW. Alcohol availability and injury: the role of local outlet densities. Alcohol Clin Exp Res 2001;25**:**1467-71

[114] Treno AJ, Johnson FW, Remer LG, Gruenewald PJ. The impact of outlet densities on alcohol-related crashes: a spatial panel approach. Accident Anal Prev 2007;39**:**894-901

[115] Trolldal B. An investigation of the effect of privatization of retail sales of alcohol on consumption and traffic accidents in Alberta, Canada. Addiction 2005;100**:**662-71

[116] Trolldal B. The privatization of wine sales in Quebec in 1978 and 1983 to 1984. Alcohol Clin Exp Res 2005;29**:**410-6

[117] Truong KD, Sturm R. Alcohol outlets and problem drinking among adults in California. J Stud Alcohol Drugs 2007;68**:**923-33

[118] Truong KD, Sturm R. Alcohol Environments and Disparities in Exposure Associated With Adolescent Drinking in California. Am J Public Health 2009;99**:**264-70

[119] Vingilis E, McLeod AI, Seeley J, et al. The impact of Ontario's extended drinking hours on cross-border cities of Windsor and Detroit. Accident Anal Prev 2006;38**:**63-70

[120] Vingilis E, McLeod AI, Seeley J, et al. Road safety impact of extended drinking hours in Ontario. Accident Anal Prev 2005;37**:**549-56

[121] Vingilis E, McLeod AI, Stoduto G, Seeley J, Mann RE. Impact of extended drinking hours in Ontario on motor-vehicle collision and non-motor-vehicle collision injuries. J Stud Alcohol Drugs 2007;68**:**905-11

[122] Waller MW, Iritrani BJ, Christ SL, et al. Relationships among alcohol outlet density, alcohol use and intimate partner violence victimization among young women in the United States. J Interpers Violence 2012;27**:**2062-86

[123] Waller MW, Iritrani BJ, Christ SL, et al. Perpetration of intimate partner violence by young adult males: The association with alcohol outlet density and drinking behavior. Health and Place 2013;21**:**10-9

[124] Waller MW, Iritrani BJ, Flewelling RL, et al. Violence victimization of young men in heterosexual relationships: Does alcohol outlet density influence outcomes? Violence Vict 2012;27**:**527-47

[125] Weitzman ER, Folkman A, Folkman MP, Wechsler H. The relationship of alcohol outlet density to heavy and frequent drinking and drinking-related problems among college students at eight universities. Health Place 2003;9**:**1-6

[126] Wicki M, Gmel G. Hospital admission rates for alcoholic intoxication after policy changes in the canton of Geneva, Switzerland. Drug Alcohol Depen 2011;118**:**209-15

[127] Wiebe DJ, Guo W, Allison PD, et al. Fears of violence during morning travel to school. J Adolesc Health 2013;53**:**54-61

[128] Wilkinson C, Livingston M. Distances to on- and off-premise alcohol outlets and experiences of alcohol-related amenity problems. Drug Alcohol Rev 2012;31**:**394-401

[129] Xu Y, Yu Q, Scribner R, et al. Multilevel spatiotemporal change-point models for evaluating the effect of an alcohol outlet control policy on changes in neighborhood assaultive violence rates. Spatial and Spatio-temporal Epidemiology 2012;3**:**121-8

[130] Young R, MacDonald L, Ellaway A. Associations between proximity and density of local alcohol outlets and alcohol use among Scottish adolescents. Health and Place 2013;19**:**124-30

[131] Yu Q, Scribner R, Carlin B, et al. Multilevel spatio-temporal dual changepoint models for relating alcohol outlet destruction and changes in neighbourhood rates of assaultive violence. Geospatial Health 2008;2**:**161-72

[132] Zhao J, Stockwell T, Martin G, et al. The Relationship between Minimum Alcohol Prices, Outlet Densities and Alcohol Attributable Deaths in British Columbia 2002 to 2009. Addiction 2013;108**:**1059-69

[133] Zhu L, Gorman DM, Horel S. Alcohol outlet density and violence: a geospatial analysis. Alcohol Alcoholism 2004;39**:**369-75
